# Influence of the visuo-proprioceptive illusion of movement and motor imagery of the wrist on EEG cortical excitability among healthy participants

**DOI:** 10.1371/journal.pone.0256723

**Published:** 2021-09-02

**Authors:** Salomé Le Franc, Mathis Fleury, Camille Jeunet, Simon Butet, Christian Barillot, Isabelle Bonan, Mélanie Cogné, Anatole Lécuyer

**Affiliations:** 1 Rehabilitation Medicine Unit, University Hospital of Rennes, Rennes, France; 2 Hybrid Team, Inria, University of Rennes, Irisa, UMR CNRS 6074, Rennes, France; 3 Empenn Unit U1228, Inserm, Inria, University of Rennes, Irisa, UMR CNRS 6074, Rennes, France; 4 CLLE Lab, CNRS, Univ. Toulouse Jean Jaurès, Toulouse, France; Anglia Ruskin University, UNITED KINGDOM

## Abstract

**Introduction:**

Motor Imagery (MI) is a powerful tool to stimulate sensorimotor brain areas and is currently used in motor rehabilitation after a stroke. The aim of our study was to evaluate whether an illusion of movement induced by visuo-proprioceptive immersion (VPI) including tendon vibration (TV) and Virtual moving hand (VR) combined with MI tasks could be more efficient than VPI alone or MI alone on cortical excitability assessed using Electroencephalography (EEG).

**Methods:**

We recorded EEG signals in 20 healthy participants in 3 different conditions: MI tasks involving their non-dominant wrist (MI condition); VPI condition; and VPI with MI tasks (combined condition). Each condition lasted 3 minutes, and was repeated 3 times in randomized order. Our main judgment criterion was the Event-Related De-synchronization (ERD) threshold in sensori-motor areas in each condition in the brain motor area.

**Results:**

The combined condition induced a greater change in the ERD percentage than the MI condition alone, but no significant difference was found between the combined and the VPI condition (p = 0.07) and between the VPI and MI condition (p = 0.20).

**Conclusion:**

This study demonstrated the interest of using a visuo-proprioceptive immersion with MI rather than MI alone in order to increase excitability in motor areas of the brain. Further studies could test this hypothesis among patients with stroke to provide new perspectives for motor rehabilitation in this population.

## Introduction

Vibratory stimulation is already used in various medical applications, such as pain management or proprioceptive rehabilitation after stroke [1,2]. It has a powerful proprioceptive role [3] and when applied under strict conditions (frequency of 80–100 Hz, tendon target) [4], it can create illusions of movement also named kinesthetic illusions (or tendon vibration-inducing illusion) [5] by apparently stimulating the brain motor areas [6]. There is a wide range of haptic stimulations, such as standard vibration, tendon vibration as described here, and pressure stimulation. Each tool has different effects on the sensory stimulation and the brain activity it triggers. In the literature on tendon vibration, we know that this kind of vibration could correspond to passive movements in terms of cortical excitability in sensorimotor areas [7]. Vibratory sensation applied to a tendon triggers the activation of local mechano-receptors, which induces a visible elongation of the tendon. This phenomenon elicits a kinesthetic illusion antagonistic to the vibrated tendon and leads to greater cortical activity in the sensorimotor motor areas and a reinforcement of activation in the propriomotor loop [8–10]. The main advantage of this propriomotor loop is that it is more effective than the visuomotor loop, which is slower and less automatic in terms of neuronal activation [11]. This vibratory mode presents 3 main advantages in the area of motor rehabilitation: the production of a kinesthetic illusion (probably through the stimulation of the motor areas), the strengthening of the sensorimotor loop, and a faster action than the visuomotor loop. It could be helpful for motor rehabilitation after neurological impairments where attention, cognitive and visual disorders can disturb the rehabilitation program.

In the last twenty years, a growing number of studies have taken an interest in developing Virtual Reality tools (VR) in various fields, such as social psychology [12], haptic technologies [13] and rehabilitation [14,15]. Virtual reality immersion is the perception of being physically present in a non-physical world. The user can interact with a virtual environment that looks realistic enough to enable a feeling of immersion [16]. The role of embodiment in VR seems valuable to immerse participants in a controlled environment and create kinesthetic illusions [17]. Combining a VR interface with haptic devices tends to increase the feeling of embodiment described in the literature, by giving a congruent tactile feedback from a visual immersive environment [18]. Rinderknecht and al. (2013) also proved that the addition of VR enhanced the perception of the illusory movement induced by tendon vibration among healthy participants [19]. Other studies have demonstrated the interest of using a virtual environment congruent with the movement of the limb to enable a better illusion and feeling of embodiment [20,21], or have demonstrated the effect of the combination of visuomotor and visuotactile stimulation on the illusion of ownership of the virtual body [22].

Another tool that can be used to stimulate motor areas of the brain is Motor Imagery (MI), which consists in imagining moving the limb without performing any actual movement. MI is already used for upper or lower limb rehabilitation in motor rehabilitation after neurological impairments [23,24]. It is now well known that MI triggers brain structures sharing similar neural networks with motor execution, including the premotor, supplementary motor, cingulate, and parietal cortical areas, the basal ganglia, and the cerebellum [25,26]. There are 2 main types of MI: the so-called visual MI (observing another person) or the so-called kinesthetic MI (observing oneself). Kinesthetic MI consists in performing the task mentally with the sensation of movement (motor and sensory). However, this kind of MI is more difficult to elaborate than visual MI. It has however been shown to activate the same neural networks as real movements in functional imagery [27] and is therefore preferred in rehabilitation. Associated with MI or used alone, Action Observation (AO) tasks have also shown their interest for activating the motor areas of the brain [28,29]. These articles prove that watching one’s own limb or another person’s limb can improve motor learning and motor skills among both healthy participants and neurological patients.

In the literature, cortical activations have been evidenced using functional imaging and electroencephalography (EEG) recordings. EEG activity can be recorded during MI or tendon vibration from electrodes placed in the motor areas of the brain. It is then processed to extract relevant features from the recorded signals. Sensory-Motor Rhythms (SMRs) consist in brain wave oscillations generated by the somatosensory and motor cortices and recorded, mainly, from the C3 (left motor area) and C4 (right motor area) electrodes. SMRs are detectable at frequencies of 8–28 Hz [30,31]. SMR rhythms are represented by Event-Related Desynchronizations (ERD) and Event Related Synchronizations (ERS), which correspond to the attenuation or increase of the strength of spontaneous EEG signals in the μ band (8–13 Hz) and β band (13–28 Hz), observed around the motor cortex in synchrony with the intention and/or execution of a MI task [32]. To summarize, SMRs are modified when performing passive and active motor movements as well as when preparing and imagining the movement [33].

The effects of the combined association of illusion of movement and MI to stimulate cerebral motor areas have not been clearly described to date. The literature suggests some hypotheses, such as the fact that proprioceptive illusions and MI could share the same neural underlying mechanisms [34]. Chatterjee and al. (2007) in a Brain-Computer Interface (BCI) study, found some interesting results on an enhancement of μ rhythm desynchronization when a vibratory actuator was applied simultaneously to the upper limb concerned by the MI, but this study did not concern vibrations with illusion of movement [35]. Recently, Yao and al. (2015) in a study on healthy participants demonstrated that tendon vibration inducing an illusion of movement significantly increased the detection accuracy of MI tasks when the vibration was applied just before the MI [36]. Finally, Barsotti and al. (2018) demonstrated that MI combined with tendon vibration seemed to increase the ERD threshold in the motor areas in the context of BCI training among healthy participants [37]. We used tendon vibration associated with a VR environment in our research: first to obtain a potentiation of the illusion, and second because the observation of a motor task plays a role in motor activations in the brain.

We hypothesized that a visuo-proprioceptive stimulation (including tendon vibration with illusion of movement and VR visual environment) combined with MI could increase the cortical excitability of brain motor areas compared to MI or visuo-proprioceptive stimulation taken separately, by the production of a virtuous closed-loop feedback. Improving and reaching the best possible cortical excitability could enable optimal brain plasticity and could thus be an efficient means of neuro-rehabilitation for patients.

The aim of our study was therefore, using EEG, to evaluate whether a visuo-proprioceptive immersion (VPI) including tendon vibration (TV) and Virtual hand movement (VR–virtual reality) combined with MI tasks could be more efficient than VPI alone or MI alone, in terms of cortical excitability of motor areas of the brain among healthy participants.

## Materials and methods

### Study design

**Fig 1:**
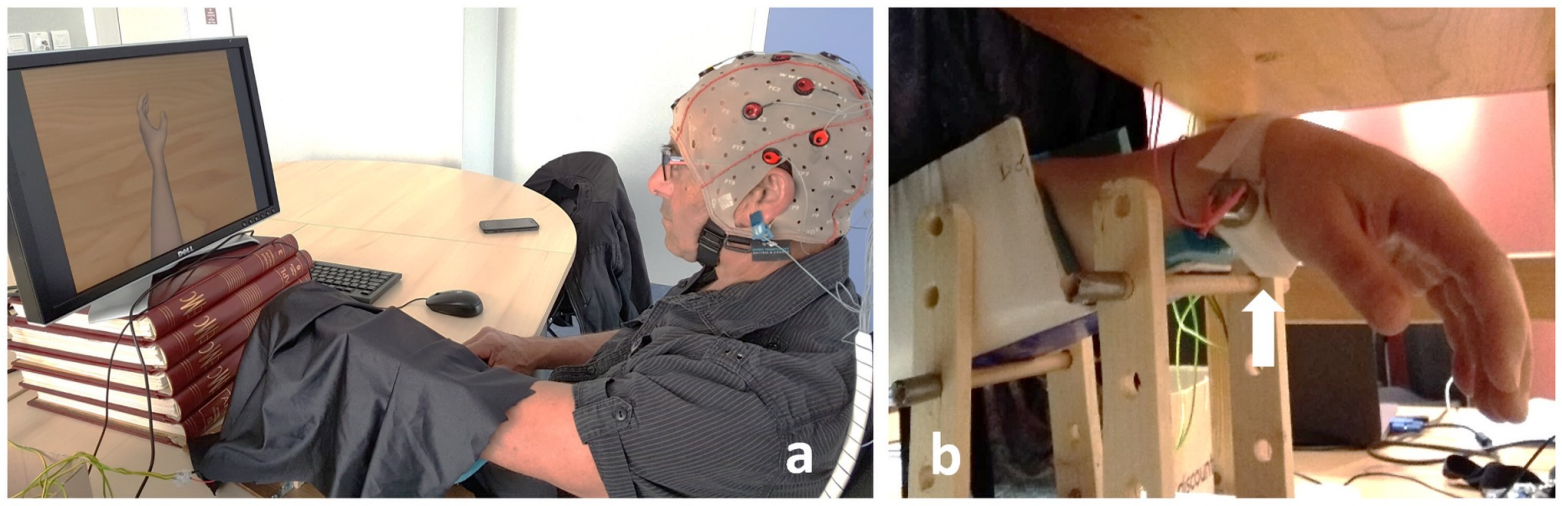
Apparatus used in the experiment. a) Positioning of the participant during EEG recording. The vibrator was positioned on the left, non-dominant wrist, hidden from view by the participant with a black cloth. b) Set-up of the vibrator on the flexor carpi tendon. The forearm was positioned in a shell. The white arrow indicates the vibrator.

From October to November 2019, we conducted a single-centre randomized controlled pilot study in the Rehabilitation Unit of Rennes University Hospital in France. The study was promoted by the Rennes University Hospital Center and obtained the approval of the Ethics Committee of Strasbourg University, France, on October 8th, 2019 (registration number: 19/62-SI 19.07.05.46737). An information letter was issued to the participants including: the aims of the study, the protocol, the risks involved and insurance details. Written consent was obtained from each participant prior to testing. This study was recorded in Clinical Trials under the following registration number NCT04130711. No changes to the study design were made after approval by the ethics committee. The individual pictured (in Fig 1a) has provided written informed consent (as outlined in PLOS consent form) to publish their image alongside the manuscript.

### Participants

Volunteering healthy participants were recruited using a public advertising strategy in the Department of Rehabilitation unit of Rennes University Hospital and of the Medicine Department of Rennes University. A total of 20 healthy participants (Mean± Standard Deviation): 31.30±9.86 years old, Median = 30, Min = 22, Max = 61, Q1 = 24.75, Q3 = 33.25 participated in the study, with 11 males (55%) and 9 females (45%). All healthy participants met the following inclusion criteria: age between 18 and 80 years; no previous history of neurological illness (brain injury, brain surgery, epilepsy), right-handed. Participants deprived of freedom and with a legal incapacity were excluded from the study. We made this protocol as a pilot study. Concerning the number of participants, the corresponding literature motivating the research hypothesis includes studies involving 15 [29] or 16 participants [37].

### Experimental procedure

**Fig 2:**
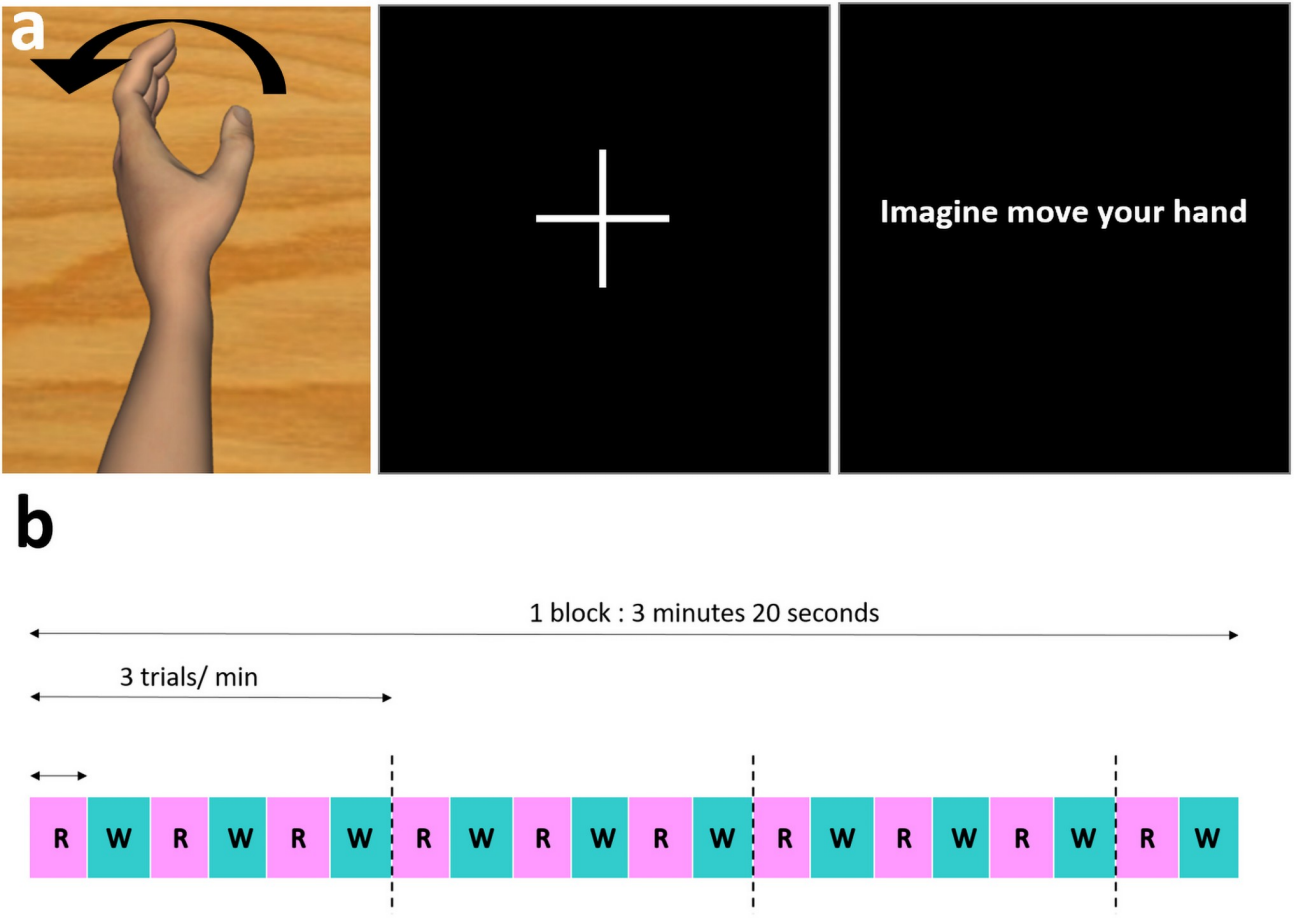
Description of the recorded sessions. **a)** Visualization of the condition on the screen: a virtual moving hand during vibration (combined condition and VPI condition), or a cross on the screen for a resting period, or a visual instruction on the screen to perform motor imagery tasks (MI condition). **b)** Descriptive diagram of one round. "R » means resting period and "W" means indifferently visuo-proprioceptive stimulation, Motor Imagery or combined stimulation.

**Fig 3:**
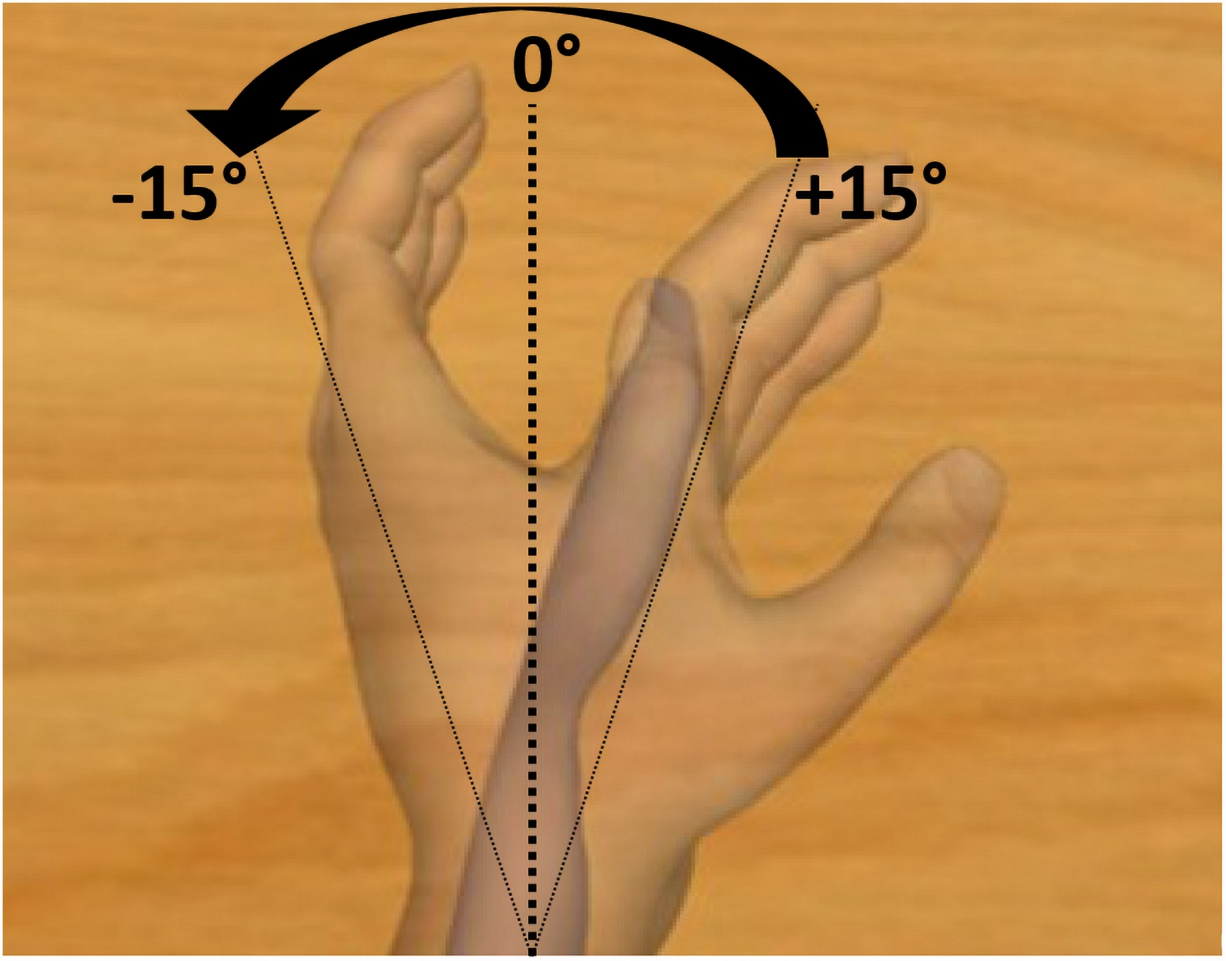
Description of the virtual cue. Virtual movements from wrist flexion to wrist extension, with a total displacement of 30 degrees around the resting position. The values and arrows were not visible to the participant during the experiment. https://doi.org/10.1371/journal.pone.0242416.g002.

The participants sat in a typical office chair in front of a computer screen in a quiet room. The EEG cap was positioned on their scalp and the vibratory mechanism on the flexor carpi tendon of their non-dominant arm (left arm), positioned in a shell and hidden from view (Fig 1a and 1b). This experiment was performed on the non-dominant left limb, because the findings of a previous study showed that the illusion of movement was greater when the tendon vibration was applied to the non-dominant limb [38]. Laterality was determined verbally and then checked using the Edinburgh questionnaire. This experiment was composed of 3x3 randomized conditions of 3 minutes 20 seconds each, and we recorded the EEG signals of the participant in each session of 3’20 minutes without any additional feedback. The first condition consisted of MI tasks (MI condition). The second condition (VPI condition) consisted of applying a wrist tendon vibration to the wrist of the non-dominant arm of the participant while he was seeing a virtual moving hand, the movement of which was congruent with the illusion of movement induced by the vibrator. The third condition combined the MI condition with the VPI condition (Combined condition) (Figs 2a and 3). Each set of MI tasks or VPI lasted 10 seconds and was separated from the next one by 10 seconds of rest. Each set was repeated 10 times to complete one session of 3 minutes and 20 seconds (Fig 2b). In the condition with MI, the instructions given to the participants were to imagine a movement similar to the one they had seen on the screen.

After the MI tasks (in combined or MI condition), a Likert scale from 1 to 7 (1 = easy, 7 = difficult) appeared on screen for the participant to quantify the difficulty in performing the MI tasks. At the end of the experiment, all the participants completed a questionnaire to determine whether they had already tried vibrating devices or MI of this type, to obtain subjective data on vibration comfort, and to obtain information about the illusion of movement felt. We then analysed the ERD values for each condition in order to determine which one was the best to improve cortical excitability and whether visuo-proprioceptive stimulation could trigger motor area activation during MI.

After VPI or the combined condition, a Likert scale from 1 to 7 appeared on screen for the participant to quantify the intensity of the illusion (1 = no illusion at all; 4 = moderate intensity of illusion of movement; 7 = strong intensity of illusion of movement).

### Visual scene

The virtual scene was shown to the participants by using Unity 3.5 software and was composed of a moving, homemade, neutral, white-skinned hand avatar. The scene was displayed on a 17 inch-LCD monitor, the angle of view was that of the virtual avatar and the monitor was positioned in order to match the participant’s first perspective. The movement executed by the virtual hand was an extension of the non-dominant wrist with a total displacement of 30 degrees from resting position, at a speed of 3 degrees per second, congruent with the illusory movement that was expected by the application of a flexor carpi tendon vibration (Figs 2a and 3) [7]. This movement matched with the illusory sensation of felt from the vibration.

### Vibratory device

**Fig 4:**
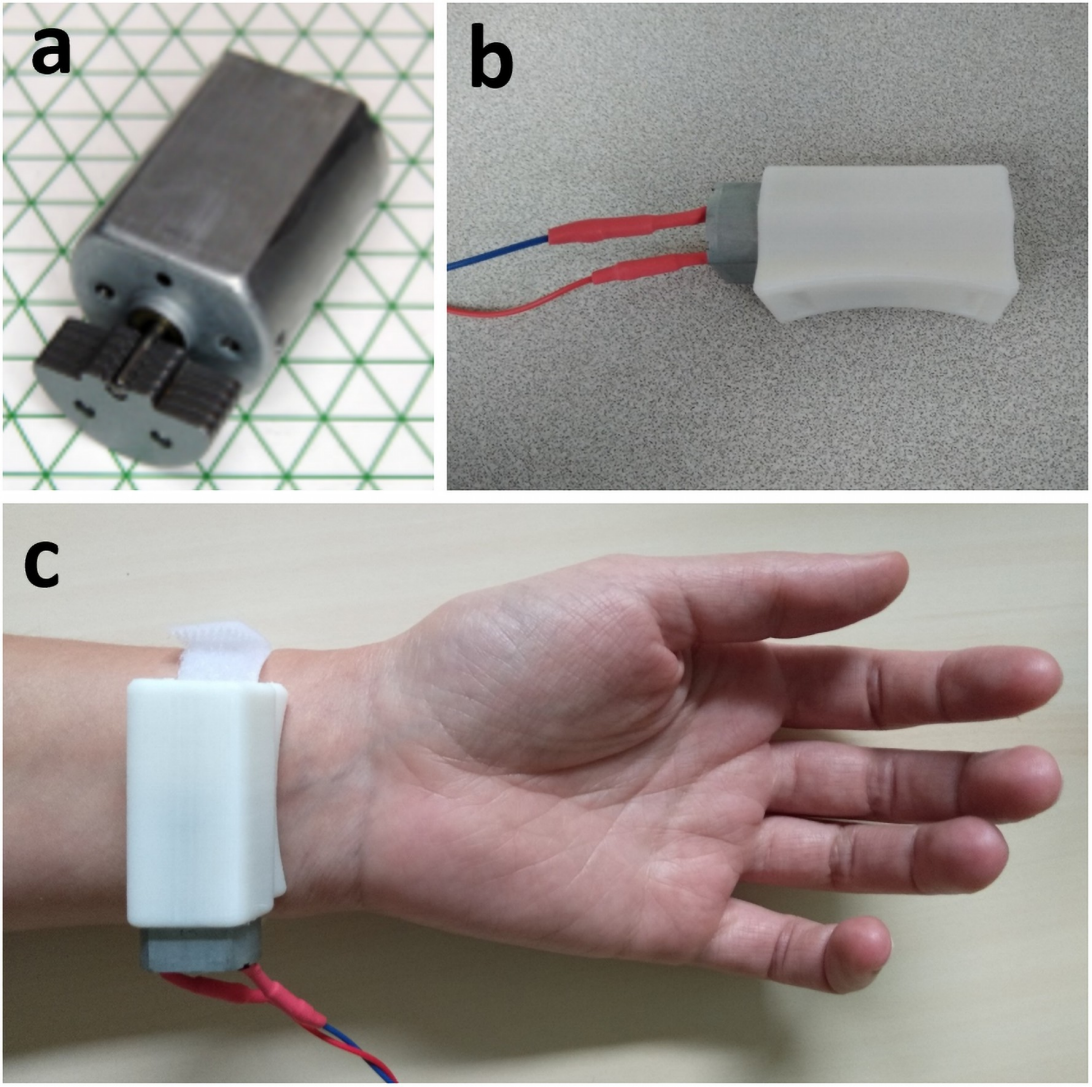
Images of the UniVibe^™^ vibratory device. **a)** Raw vibration motor. **b)** Vibration motor device linked to the Arduino^®^ and inside a sound box. **c)** Wrist placement. https://doi.org/10.1371/journal.pone.0242416.g003.

The device used in this work was a UniVibe^™^ Model 320–105 vibratory unit (Fig 4), which is composed of an actuator with an adjustable position and orientation that can be finely positioned on the flexor carpi tendon and maintained on the skin with hook-and-loop fastener. We created a sound box by 3D printer to protect the skin from the motor and to enhance the sensation of vibration. An Arduino^®^ controlled the vibration motor. The vibration frequency is determined by the rotation of the mass. The diameter of the skin actuator is 25 mm. In this study, we applied a frequency of 100 Hz, an amplitude of 5G, and voltage of 3.3 V on the basis of [4,38,39] to elicit an illusion of movement. We explained orally to the participants that they would receive bursts of vibration on their wrist, which might give them a feeling of movement. We did not specify what movement it might be (hand, finger…) nor in which direction it would occur.

### EEG data acquisition

We recorded EEG data in an extended 10–20 system with g.Tec^®^ cap and g.Amp amplifier. The data were processed using a customised processing algorithm on OpenViBE software. A pattern of 16 electrodes was placed over the scalp: Fpz, Fz, Cz, Pz, Fc1, Fc2, Fc5, Fc6, C3, C4, T7, T8, Cp1, Cp2, Cp5, Cp6. The ground electrode was placed on AFz and all channels were referenced to the right ear lobe electrode.

### EEG data analysis

The EEG analysis was performed on electrodes Cz, Fc1, Fc2, Fc5, Fc6, C3, C4, Cp1, Cp2, Cp5, Cp6 (relating to the motor areas). EEG recordings were band-pass filtered from 0.5 to 40 Hz and then digitally converted with a sample frequency of 512 Hz (using a Butterworth zero phase filter with a 48 dB slope). All EEG recordings were inspected visually and electrodes with too high impedance (>30 kHz) or poor signal quality were excluded from further analysis. Concerning the 20 subjects in the study, 16 of them have intact sessions and 4 of them have one session deleted (one third of a condition). Imagined movement in sensorimotor rhythm paradigms causes ERD in μ (8–13 Hz) and β (13–28 Hz) rhythms, known to be involved in imagining movement, task preparation or active tasks, and observed in the primary motor cortex, contra-lateral to the limb involved in the task, mainly related to C3 and C4 electrodes [30]. The ERDs were extracted at a Riemannian distance [40]. We did not include a spatial filter in the analysis, instead we directly observed the entire signal strength of the electrodes. This relative power change is calculated according to:

$$\%{Power}_{E}=\frac{{task}_{E}-{rest}_{E}}{{rest}_{E}}$$

With *task*_*E*_ and *rest*_*E*_ denoting the average power in the frequency range of electrode E during the task condition and at rest, respectively. Positive power changes in strength will be referred to as ERS whereas negative changes will be referred to as ERD. ERD and ERS are percentage values, the reference value being calculated according to the remaining tasks. Results were visualized for the alpha (8–13 Hz) and beta (13–28 Hz) bands as topoplot maps.

### Data collected

The primary outcome measure was the ERD values measured in μ and β bands on the C4 electrodes in each condition (MI condition, VPI condition, combined condition). We used C4 electrode as primary outcome because it was estimated that the most important information would come from the right brain motor areas. We then examined other electrodes near C4. The secondary outcome measures were: a) the intensity of the illusion of movement felt during tendon vibration scored on a Likert scale (from 1 to 7 with 1 = no illusion at all; 4 = moderate intensity of illusion of movement; 7 = strong intensity of illusion of movement) and b) the difficulty in performing MI tasks (from 1 to 7 with 1 = easy to 7 = difficult). The participants had a computer mouse (with their free right hand) which enabled them to respond on the Likert scale on the screen after each set. The data were collected in the Data Archiving and Networked Services (DANS) databases.

### Software and statistical analysis

Data processing and signal analysis were performed using MATLAB R2017a (MathWorks, Inc., Natick, MA, United States). The results for the spatial activation patterns are given as the relative power change from baseline ± standard deviation (SD). Topographical plots were created using a customised MATLAB function. We used percentages to describe qualitative variables, and mean ± standard deviation to describe quantitative variables for parametric data. We also used median and interquartile intervals to describe non-parametric data. All data were analyzed by statistical tests using R and MATLAB software. According to the Shapiro-Wilk test, our data followed a normal distribution (p = 0.25, p = 0.73, p = 0.11 respectively for the combined condition, for the VPI condition and for the MI condition). According to Mauchly’s Test of Sphericity, our data set violated the assumption of sphericity, for the main judgment criterion: χ^2^ = 24.47, p<0.001. We therefore used a non-parametric approach (Friedman test and then Wilcoxon signed rank test) to compare mean ERDs on C3 and C4 electrode across condition. We used a two-way repeated ANOVA to compare each conditions (MI condition, VPI condition, combined condition) in each band frequency (μ band, ß band, μ + ß bands) for all the participants and we tested for an interaction between condition and frequency, then we used a Kruskal-Wallis test to compare the mean ERDs according to subgroups. We also used a Wilcoxon rank sum test to compare data between two conditions. P-values <0.05 were considered statistically significant.

## Results

**Fig 5:**
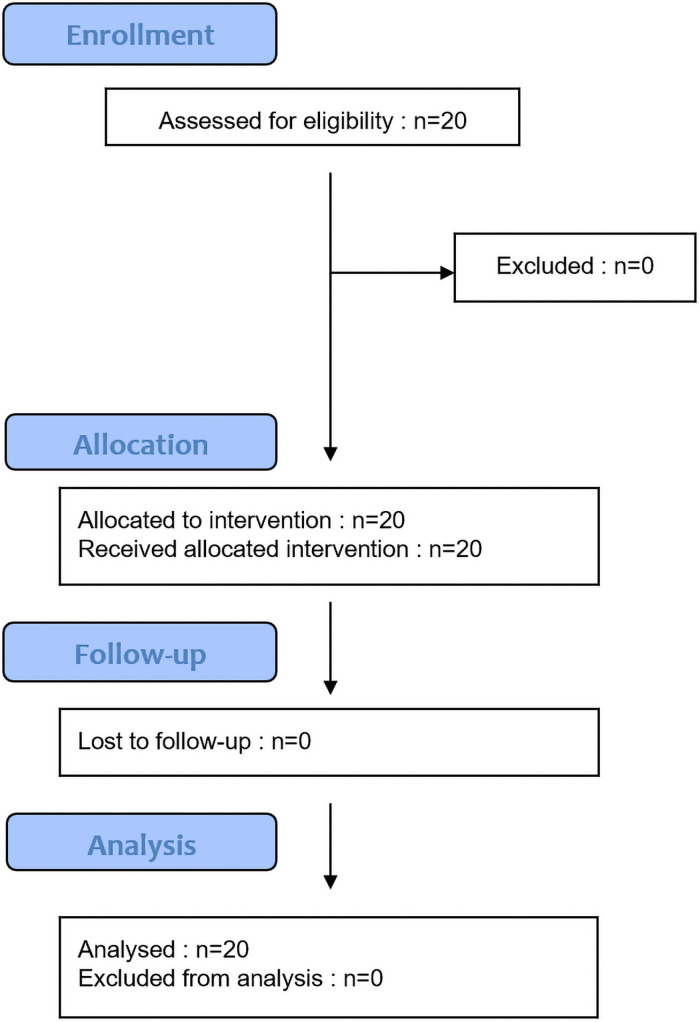
Flowchart of the experiment.

This is the flowchart of the experiment (Fig 5).

### EEG data

**Fig 6:**
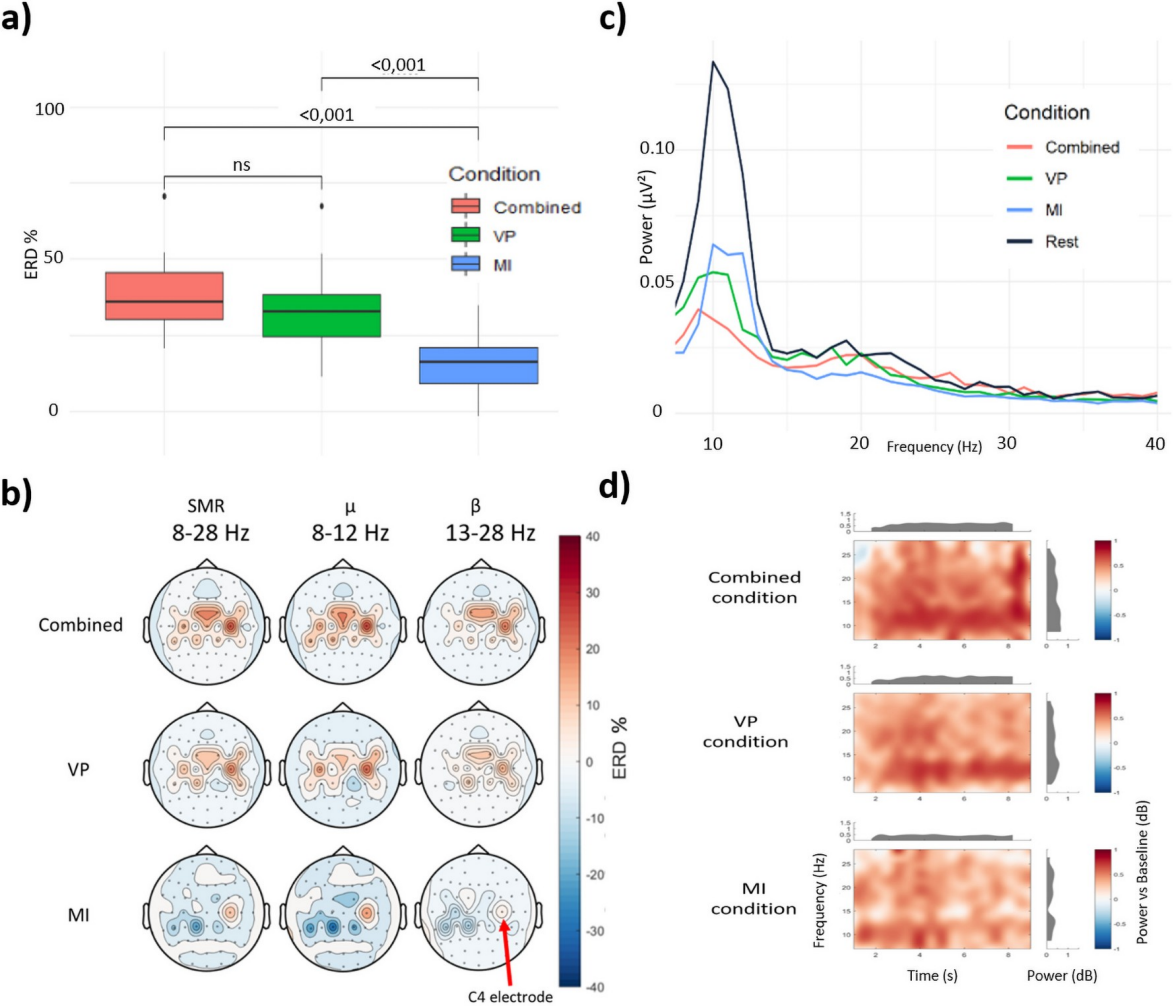
EEG results. ***a) Boxplot of ERD percentages measured in each condition in the 8–28 Hz bands***. The left boxplot represents the ERD percentage on the average of the μ and β frequencies in the combined condition (“Combined”), the second boxplot concerns the VPI condition (VP), the right boxplot concerns the MI condition (M). The crosses represent the means for ERD. NS means “non significant”. ***b) Topoplots of ERD percentages measured in each condition***. The topoplots were averaged across all the participants. They are separated into rows, according to each condition tested (combined condition, VPI condition (VP), motor imagery condition (MI)), and in columns for 8-28Hz (μ-β bands), 8-13Hz (μ bands), and 13-28Hz (β bands). The red arrow locates the position of the C4 electrode. Red represents ERD; blue represents ERS. ***c) Power spectrum density analysis***. Representation of each condition as a function of the signal power in the 8–28 Hz frequency bands on the C4 electrode. Each line represents one condition. Black: Resting state, red: combined condition, green: VPI condition (VP), blue: MI condition. ***d) Time frequency analysis***. Representation of signal power as a function of task completion time (10 seconds) in the 8–28 Hz frequency bands under each condition. From top to bottom: combined condition, VPI condition (VP), MI condition. The red colour represents a greater decrease in signal power.

The mean ERD (C4 electrode) values in percentages ± Standard Deviation were 38.00±10.08 for the combined condition, 33.04±9.93 for the VPI condition and 13.34±12.08 for the MI condition (Fig 6a). Using a Friedman test, there were significant differences across the 3 conditions (χ^2^ = 24.47, p<0.001) according to the ERD percentage. There was no significant difference between the combined condition and the VPI condition (p = 0.59), but there was a significant difference between the combined condition and the MI condition (p<0.001) and between the VPI condition and the MI condition (p<0.001).

The mean ERD (on C3 electrode) values were 19.88±12.16 for the combined condition, 7.06±23.88 for the VPI condition and -3.67±28.89 for the MI condition. Using a Friedman test, there were significant differences across the 3 conditions (χ^2^ = 17.20, p<0.001) according to the ERD percentage. There was a significant difference between the combined condition and the VPI condition (p<0.01), between the combined condition and the MI condition (p<0.001) but there was no significant difference between the VPI condition and the MI condition (p = 0.06).

We then performed analyses using a two-way repeated ANOVA in order to consider the different conditions and each frequency band. We found that the combined condition was significantly superior to the MI condition (F = 14.84, p<0.01) and we also found a significant result depending on the frequency band (F = 10.05, p<0.01). Nevertheless, we did not find significant differences by comparing the VPI and MI condition on C4 electrode using this method (F = 1.83, p = 0.20 and F = 0.34, p = 0.57) for the conditions analysis nor for the frequencies analysis. We have also tested for an interaction between frequency and condition. We performed the test by comparing each condition to the others (i.e, MI and VPI condition, MI and combined condition, VPI and combined condition). There was no significant difference in the results: between the MI condition and VPI condition (Anova, F = 1.93, p = 0.19), between the MI condition and combined condition (Anova, F = 0.19, p = 0.67), between the VPI condition and combined condition (Anova, F = 2.66, p = 0.13). Then, by using a Kruskal-Wallis test, we found that the combined condition was significantly superior to the MI condition in the μ band, ß band and μ + ß band for all the brain motor electrodes (C4, CP2, FC2, CP6, FC6). Comparing the VPI and MI condition, we found only a significant difference concerning the FC2 electrode. Similarly to our previous analyses, we did not find any significant difference between the combined condition and the VPI condition.

According to the data, the topoplots represented the power of ERD measured in the 8–28 Hz bands (μ and β bands) around the scalp averaged across all the participants in the study (Fig 6b).

Concerning C4 electrode, among the participants who preferred to perform the MI tasks during vibration (n = 9), the ERD average value was 41.85% ± 11.11, while in the population of participants who found MI more difficult to perform when visuo-proprioceptive stimulation was present at the same time (n = 11), the result was 34.86% ± 8.41. There was no significant difference between the two groups (Wilcoxon rank sum test, W = 63, p = 0.33). The groups were defined following the subjective reports. The participants evaluated the difficulty to perform MI during tendon vibration by using a Likert scale from 1 (very difficult) to 5 (easy to perform).

Among the 5 participants who had previously performed MI tasks (see subjective reports), the ERD value was 5.82% ± 27.45 in the MI condition, differing from the other participants who had never performed it before 16.05% ± 6.94. There was no significant difference between the groups (Wilcoxon rank sum test, W = 40, p = 0.87).

We did not find any correlation between the intensity of the illusion of movement felt by the participants and the level of cortical activation represented by the ERD in the VPI condition (Pearson’s test, r = -0.08, p = 0.72) or in the combined condition (r = -0.07, p = 0.77).

A power spectrum density (PSD) analysis showed the evolution of the signal power according to the frequency bands of interest in the 8–28 Hz range on the C4 electrode, according to each condition and in comparison with the resting state (Fig 6c). There were significant differences across the 3 conditions (χ^2^ = 12.77, p<0.01) according to the PSD values. There was no significant difference between the combined condition and the VPI condition (p = 0.24), but there was a significant difference between the combined condition and the MI condition (p<0.01) and between the VPI condition and the MI condition (p<0.001). We compared the values in each condition between the task and the corresponding resting period: we found a significant difference in each condition: Combined condition, VPI condition and MI condition (p<0.001).

A time-frequency analysis was performed for each condition, with the average results of all study participants averaged over the 10-second period of stimulation in the 8 to 28 Hz frequency bands (Fig 6d).

### Intensity of the illusion of movement

The median (Q1, Q3) for Likert ratings was respectively 6 (4, 6) for the VPI condition and 5 (4, 6) for the combined condition. There was no significant difference across conditions (Wilcoxon signed rank test, p = 0.85, W = 501).

**Fig 7:**
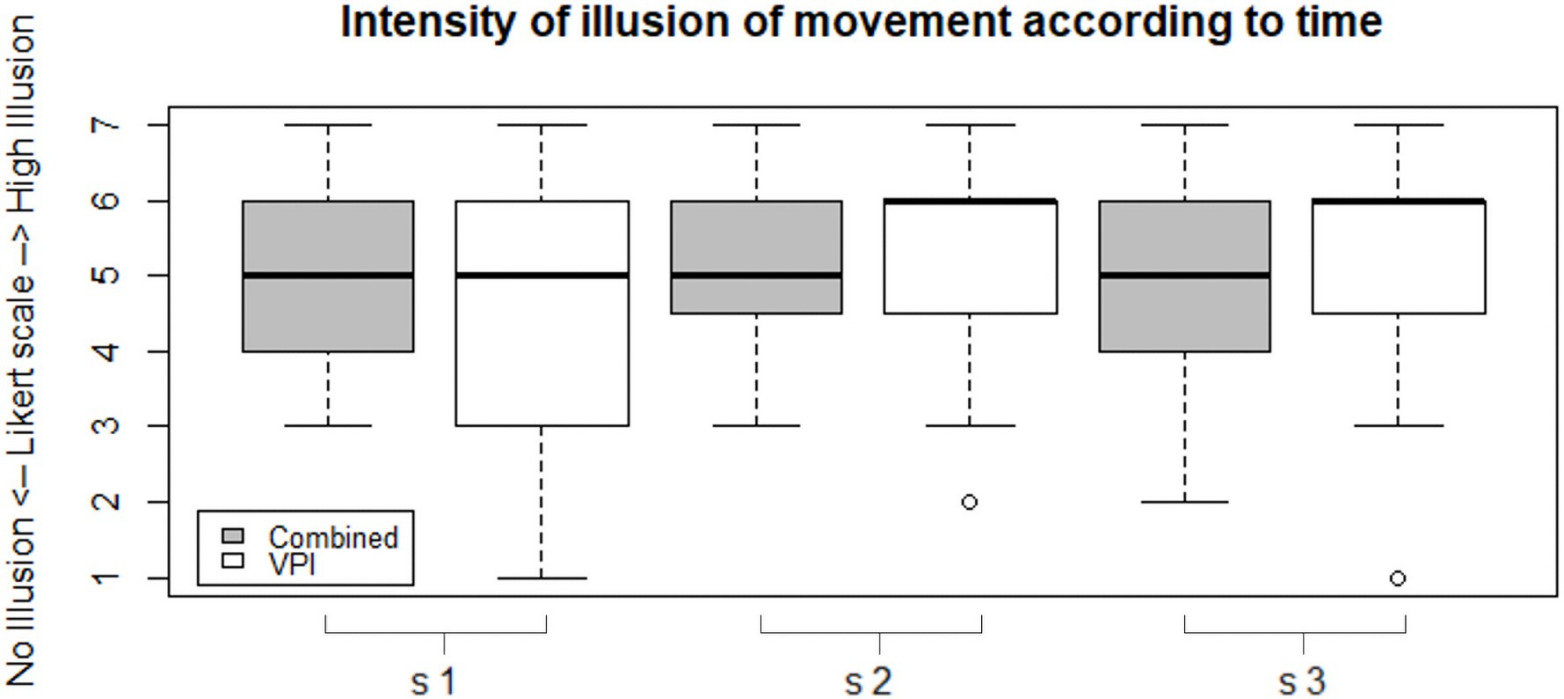
Intensity of illusion of movement according to time. Boxplot representations of the intensity of illusion of movement in combined condition (grey boxplot) and in VPI condition (white boxplot) according to time (session 1 (s1) to 3 (s3)).

The intensity of illusion of movement was also measured across time according to the Likert ratings. For each condition, we analyzed the Likert scores measured at the start of the experiment compared to the values at the end of the experiment. The median (Q1, Q3) was 5 (3, 6) (in session 1 of the condition i.e. the first 3 recorded minutes) and then 6 (4.75, 6) (in session 3 of the condition i.e. the last 3 recorded minutes) for the VPI condition. There was no significant difference between sessions 1 and 3 (Wilcoxon signed rank test, p = 0.30, W = 47.5). The median (Q1, Q3) was 5 (4, 6) (in session 1 i.e. the first 3 recorded minutes) and then 5 (4, 6) (in session 3 i.e. the last 3 recorded minutes) for the combined condition (Fig 7). There was no significant difference between sessions 1 and 3 (Wilcoxon signed rank test, p = 0.67, W = 59.5).

### Perceived ability to perform the MI tasks

The median (Q1, Q3) Likert ratings were respectively 4 (3, 5) for the MI condition and 4 (3, 5) for the combined condition. There was no significant difference across conditions (Wilcoxon signed rank test, p = 0.16, W = 665).

**Fig 8:**
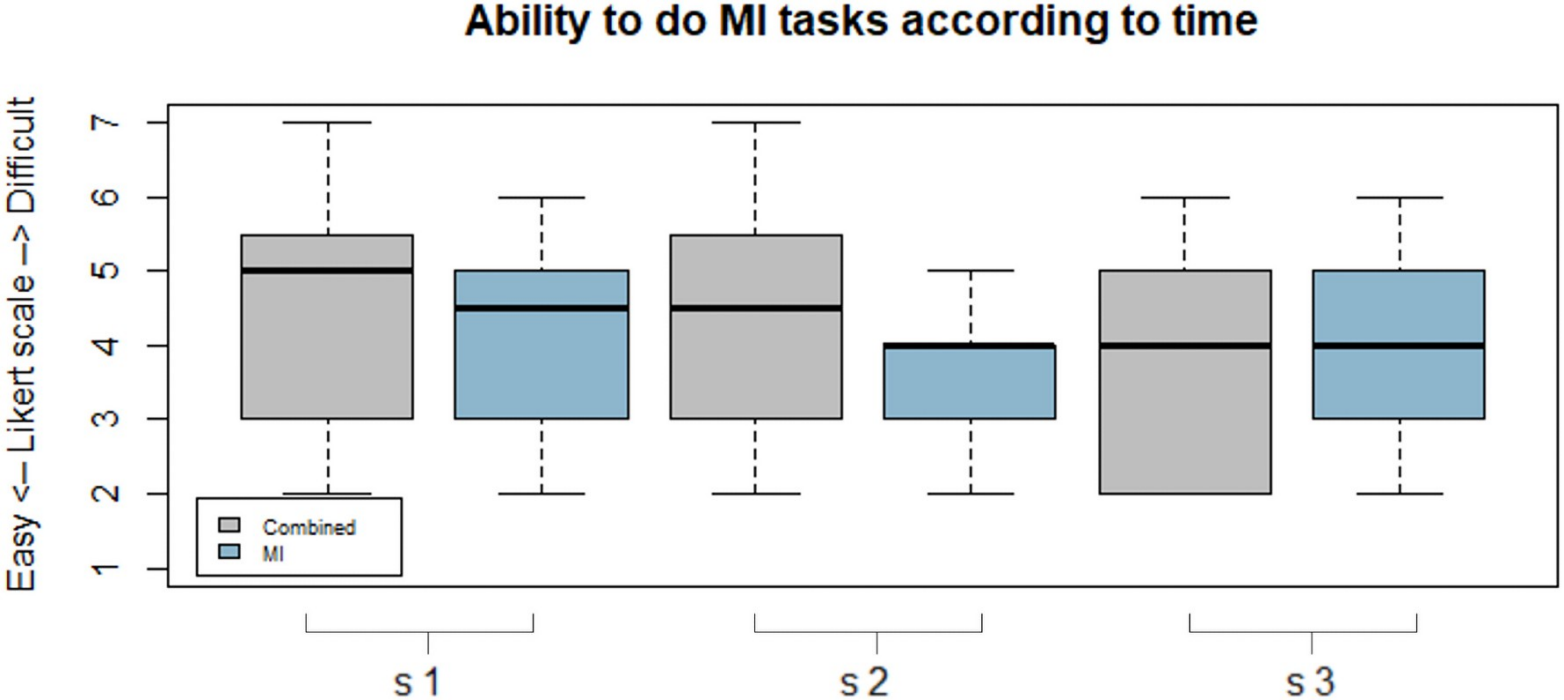
Perceived ability to perform motor imagery tasks according to time. Boxplot representation of the perceived ability to perform motor imagery tasks in combined condition (grey boxplot) and MI condition (blue boxplot) according to time (session 1 (s1) to 3 (s3)).

The perceived aptitude to perform the MI tasks was also measured according to time following the Likert rating. For each condition, we analyzed the Likert ratings measured at the start of the experiment compared to the values at the end of the experiment. The median (Q1, Q3) was 4.5(3, 5) (in session 1 of the condition i.e. the first 3 recorded minutes) and then 4 (3, 5) (in session 3 of the condition i.e. the last 3 recorded minutes) for the MI condition. There was no significant difference between sessions 1 and 3 (Wilcoxon signed rank test, p = 0.31, W = 69). The median (Q1, Q3) was 5 (3, 5.2) (in session 1 i.e. the first 3 recorded minutes) and then 4 (2, 5) (in session 3 i.e. the last 3 recorded minutes) for the combined condition (Fig 8). There was no significant difference between sessions 1 and 3 (Wilcoxon signed rank test, p = 0.19, W = 83.5).

### Subjective reports by participants

Among our 20 participants, 5 participants (25%) had already performed MI tasks at least once. Most of our participants felt tired at the end of the experiment (n = 13, 65%), but 16 participants (80%) thought that there was sufficient resting time during the protocol. Nine participants (45%) thought that their MI performance was better and easier when the tendon vibration was applied simultaneously.

## Discussion

The main aim of our experiment was to evaluate whether a visuo-proprioceptive stimulation including an illusion of movement induced by tendon vibration, a VR environment and MI tasks could be more efficient in terms of cortical excitability in sensorimotor areas than either MI tasks or visuo-proprioceptive stimulation alone.

First, the results demonstrated that the combined condition was significantly better than the MI condition in terms of ERD values for motor area of the brain. However, we did not find any significant difference between the combined condition and the VPI condition, and between the VPI condition and the MI condition, considering the two-way repeated ANOVA. In time-frequency analyses, we observed greater ERD peaks in the combined condition than in the other conditions, in the μ bands (8–13 Hz), within the stimulation time (Fig 6d). In power spectrum density graphs, we also found a greater decrease in ERD power in the combined condition than in the other conditions and relative to the resting state (Fig 6c). We could therefore hypothesize a partial synergy of the VPI and MI compared to MI alone. These results could provide interesting arguments in favour of using these tools in rehabilitation. It could be useful to use external stimulation, such as vibration, to generate greater cortical responses. The visual and proprioceptive immersion enables both sensory-motor and visuo-motor loop reinforcement. Nevertheless, an inner stimulation such as mental imagery, generated by the subject, which is introspective, controlled and more "active", is a more dynamic mode from a rehabilitative point of view. Although they are two different techniques, MI and tendon vibration (involving illusions of movement) share certain common neural substrates. Several studies using functional brain imaging have shown that similar areas were activated by these two techniques [8,23]. Other studies have also showed that one tool could interact with the other to improve it. For example, Shibata and al. (2017) demonstrated that the velocity of perceived movement was significantly greater when the participant performed MI at the same time as a vibration stimulation than with vibration stimulation alone [41].

One hypothesis that could explain the lack of evidence of superiority of the combined condition compared to the VPI condition is the possible absence of physical accumulation of ERD from different origins, and the difference in the behaviour modulation of ERD/ERS. Rimbert et al. showed that combined MI and electrical stimulation modulated the generation of ERD and ERS differently from MI alone or electrical stimulation alone. The ERDs did not seem to accumulate whereas the ERS were significantly amplified in the same condition [42]. The work by Rimbert et al. work also suggested that ERD and ERS produced by MI tasks were modulated according to the time allocated to the tasks. The MI was either "continuous" (repetitive for 4 seconds) or "discrete" (once 1000 ms). It appeared that detectable ERD/ERS in the category of discrete MI were more consistent and more easily detected than continuous MI, with ERD found to be markedly lower in continuous MI [43]. This phenomenon suggests that the ERD and ERS components overlapped over time in continuous MI [44]. We could deduce from this study that the detection of ERDs under MI conditions was not optimized by the overlapping of these repeated ERD/ERS phenomena in our study. Our ERDs were recorded from the time the starting-point was given and for a duration of 10 seconds. However, the combined condition could not have been achieved over shorter durations, since the illusion of motion induced by the vibration generally requires more than 5 seconds to appear and more than 10 seconds to be optimal [38].

In the vibration condition (Fig 6b), we found bilateral activations (ERD) over the parietal areas that were more intense on the contralateral side of the vibration. These results are in line with the literature, which has already described bilateral activations of the parietal operculum [7] and bilateral activations at the start of the execution of movement [45]. The ERD activity related to the pre-frontal cortex (FC1-FC2 electrodes) seemed to increase in both vibratory conditions. This could be explained by the stimulation of prefrontal medial regions involved in the awareness of illusory movements as described in the literature [46,47]. We also found significant ERS values over the left (ipsilateral) hemisphere due to the MI task hand. ERSs were detectable in both μ and β rhythms, in line with the current literature which reports significant ERSs on the ipsilateral side in the 5 seconds after the start of the exercise [31].

In our study, 9 participants (45%) thought that their MI performance was better and easier when the VPI was applied simultaneously with their MI tasks, while the others felt that it was disturbing. This subjective result could be explained by the positive effect of repeated tendon vibrations inducing illusory movement while the participant were performing MI tasks to enhance their MI performance, as noted by Yao and al. (2015) [36]. In this study, the participants improved their MI performances and accuracy, evaluated using EEG, by repeated tendon vibration inducing an illusion during the MI tasks. However, the ERD values were not higher for the participants who experienced facilitated MI during tendon vibration than for those who felt disturbed by tendon vibration. This could mean that the perceived ability to perform MI tasks is very subjective and cannot predict the modulation of ERD values. Our results also demonstrated the subjective difficulty of performing MI tasks for naive users (Fig 8). These data are generally in line with the literature on the topic. The participants needed to practice MI tasks many times to complete the exercise more easily [48–50].

We found that the sensation of illusion induced by tendon vibration was constant throughout the time of the experiment, without any habituation among the participants (Fig 7). By using short periods of tendon vibration in our protocol (10 seconds for each trial, repeated 60 times), we were in accordance with the current literature where short periods of vibration (10–60 sec) to induce a marked sensation of illusion of movement over the entire period tested, without habituation effect, were described [38,51].

However, our study presents some methodological limitations. First, we included a small population of 20 healthy participants. We only found in the existing literature 2 studies with a similar topic and methodology and none of them seemed to perform a power calculation [29,37]. We conceived this research as a pilot study but did not clearly explain in the first place in our manuscript how the protocol had been built. We have now made the appropriate changes in the manuscript. We used a 16-electrode cap which is still common practice in MI analysis [52] enabling an easier set-up. In the future, it might be interesting to use a cap with more electrodes enabling a more precise analysis of the sensory-motor and frontal areas.

It could be interesting in further studies to test the same hypothesis for patients with stroke to see if similar results are obtained. This population is often older [53] than the healthy participants included in our study. The elderly have more difficulty perceiving illusion of movement induced by tendon vibration [54] and we can expect a poorer illusion of movement felt by patients with stroke because some of them will also present cognitive difficulties such as attentional or sensory disorders. The current literature [55,56] remains unclear about the effectiveness of central integration of peripheral vibrations in this population.

BCI studies can use MI as a substrate, and there is a wide corpus of literature on the haptic feedback used [57]. Vibratory haptic feedback can generate artefacts in signal interpretation and it is necessary to understand how to use this feedback for more effective results and to differentiate between cortical activity from vibration and that from MI in the BCI system.

In conclusion, we found that visuo-proprioceptive stimulation and MI could be efficient to stimulate brain motor areas. VPI is an attractive and interesting tool to use in BCI systems, with MI. Overall, our results pave the way for the design of new tools in rehabilitation using VR and haptic stimulation associated with a brain-computer interface.

## Supporting information

S1 Checklist TREND statement checklist. (DOCX)
